# An engineering probiotic producing defensin-5 ameliorating dextran sodium sulfate-induced mice colitis via Inhibiting NF-kB pathway

**DOI:** 10.1186/s12967-020-02272-5

**Published:** 2020-03-02

**Authors:** Lishan Zeng, Jiasheng Tan, Meng Xue, Le Liu, Mingming Wang, Liping Liang, Jun Deng, Wei Chen, Ye Chen

**Affiliations:** 1 Department of Gastroenterology, State Key Laboratory of Organ Failure Research, Guangdong Provincial Key Laboratory of Gastroenterology, Nanfang Hospital, Southern Medical University, Guangzhou, 510515 People’s Republic of China; 2 Department of Gastroenterology, Dongguan Third People’s Hospital, Affiliated Dongguan Shilong People’s Hospital of Southern Medical University, Dongguan, Guangdong People’s Republic of China; 3 State Key Laboratory of Food Science and Technology, School of Food Science and Technology, Jiangnan University, Wuxi, Jiangsu People’s Republic of China

**Keywords:** Ulcerative colitis, Defensin-5, NZ9000SHD-5, NF-κB, Mucosal barrier

## Abstract

**Background:**

Human defensin-5 (HD-5) is a key antimicrobial peptide which plays an important role in host immune defense, while the short half-life greatly limits its clinical application. The purpose of this study was to investigate the effects of an engineering probiotic producing HD-5 on intestinal barrier and explore its underlying mechanism

**Methods:**

We constructed the pN8148-SHD-5 vector, and transfected this plasmid into *Lactococcus lactis* (*L. lactis*) to create the recombinant NZ9000SHD-5 strain, which continuously produces mature HD-5. NZ9000SHD-5 was administrated appropriately in a dextran sodium sulfate (DSS)-induced colitis model. Alterations in the wounded intestine were analyzed by hematoxylin–eosin staining. The changes of intestinal permeability were detected by FITC-dextran permeability test, the tight junction (TJ) proteins ZO-1 and occludin and cytokines were analyzed by western blotting or enzyme linked immunosorbent assay. In Caco-2 cell monolayers, the permeability were analyzed by transepithelial electrical resistance, and the TJ proteins were detected by western blotting and immunofluorescence. In addition, NF-κB signaling pathway was investigated to further analyze the molecular mechanism of NZ9000SHD-5 treatment on inducing intestinal protection in vitro.

**Results:**

We found oral administration with NZ9000SHD-5 significantly reduced colonic glandular structure destruction and inflammatory cell infiltration, downregulated expression of several inflammation-related molecules and preserved epithelial barrier integrity. The same protective effects were observed in in vitro experiments, and pretreatment of macrophages with NZ9000SHD-5 culture supernatants prior to LPS application significantly reduced the expression of phosphorylated nuclear transcription factor-kappa B (NF-κB) p65 and its inhibitor IκBα.

**Conclusions:**

These results indicate the NZ9000SHD-5 can alleviate DSS-induced mucosal damage by suppressing NF-κB signaling pathway, and NZ9000SHD-5 may be a novel therapeutic means for ulcerative colitis.

## Background

Ulcerative colitis (UC) is a chronic and recurring inflammatory bowel disease (IBD) with high prevalence worldwide, which primarily occurs in the rectal and colonic mucosa as the result multiple complex etiological interactions between genetic and environmental factors [1]. Although the pathogenesis of IBD remains unknown, immune hyperactivity, genetic susceptibility, microbiota dysbiosis and other factors have been probably directly involved in the initiation of inflammation and increased disease activity. During chronic intestinal inflammation, intestinal epithelial cells (IECs) are exposed to numerous pro-inflammatory and anti-inflammatory cytokines, which are produced by multiple immune and non-immune cells as well as by IECs themselves. For instance, pro-inflammatory cytokines, such as tumor necrosis factor-α (TNF-α) and interleukin-6 (IL-6), alter tight junction activity and contribute to apoptosis of IECs, thus leading to the loss of barrier function [2].

In the early stage of IBD, intestinal barrier is just destroyed, antigens from the intestinal cavity (*Mycobacteria, Campylobacter, Helicobacter* and *Escherichia coli*, etc.) are displaced [3]. On the one hand, the apoptosis of IECs is induced; on the other hand, the direct contact with immune cells (such as macrophages, neutrophils, dendritic cells, mastocytes, etc.) in intestinal lamina propria stimulates strong immune response, accompanied by a large number of inflammatory factors and interactions of these factors. If not effectively controlled in a short period of time, inflammatory mediators accumulated constantly, toxic effects on IECs and autoantibodies inspire autoimmune response, leading to the development of gut injury and forming a vicious circle, which is also one of the reasons why IBD often breaks out repeatedly.

Intestinal tight junctions (TJ) maintain a mucosal barrier to prevent invasion by noxious molecules, including pathogens, toxins, and antigens, while regulating permeability to ions and nutrients [4]. Disrupting the TJ barrier increases paracellular permeability to harmful pathogens and facilitates immune activity, resulting in subsequent inflammation and tissue damage [5]. TJ proteins confer the polarity of the epithelium by demarcating the cells upper and lower regions. Occludins and claudins are the core TJ proteins that control the structure and permeability of intestinal epithelium. Zonula occludens (ZO-1, ZO-2, ZO-3) are framework forming proteins connecting the actin cytoskeleton with above two transmembrane proteins [6]. As a result of either inflammation, gene mutations or an aberrant signaling transduction, TJ barrier defects disturbs the epithelial permeability and homeostasis, consequently results in the development of disease such as IBD and other intestinal barrier related abnormalities.

In patients with IBD, the typical continuous linear structure of the TJ of normal epithelial cells transformed into a broken, discontinuity granule-like appearance. At the molecular level, the expression of claudin-2 increased, which drive cations and water to enter the intestinal cavity through paracellular channels, resulting in permeable diarrhea. In addition, the excessive secretion of TNF-α, IL-6 and IL-13 was related to the increased claudin-2 expression [7, 8]. Other tight junction proteins, such as claudin-4 and claudin-7 decreased in UC, and claudin-5 and claudin-8 decreased in Crohn’s disease (CD), while the expression of occludins decreased in both UC and CD [7]. Moreover, the down-regulation of TJ proteins above has been confirmed to be related to the role of cytokines IFN-γ and TNF-a [7, 9, 10]. Previous research has shown decreased expression of TJ proteins in the intestinal mucosa of IBD patients [11]. This impaired gut barrier function in IBD suggests dysregulated TJ may participate in the pathogenesis of IBD.

Defensins are antimicrobial peptides secreted from different cells as a component of innate host defense, protecting the body from virulent pathogens such as bacteria, viruses and fungi [12]. Two classes of human defensins have been distinguished based on structural differences in their disulfide bonds: α-defensins and β-defensins. The former is secreted by Paneth cells in the intestine, playing an essential role in intestinal barrier function and homeostasis, participating in immune regulation and controlling microbiota composition. Some studies have indicated α-defensin expression is decreased in the ileal tissue of IBD patients [13, 14], but Paneth-cell-specific α-defensin-derived fragments with antimicrobial activity might be an important part of intestinal barrier function, influencing the existing microbiota by controlling important bacteria like *Akkermansia muciniphila* [15]. However, increased human defensin-5 (HD-5) expression in colonic crypts has also been reported, and additional peptides are produced in response to the severe inflammation or contribute to the onset of intestinal inflammation due to dysregulation of intestinal microbiota [16, 17]. A recent study suggest that Gram-negative bacterium *Shigella* could weaken host defense by hijacking HD-5 as an unwitting accomplice to enhance its adhesion and invasion of the host epithelium [18]. Thus, it is unclear whether these alterations in defensin expression are responsible for the pathogenesis of IBD or a byproduct of disease progression, and to date, there is little focus on therapies targeting this phenomenon. In this study, we investigated the effects of exogenous administration of HD-5 on the regulation of immunological responses and the protection of intestinal TJ in colitis. Because human defensin is primarily extracted from natural resources and as its synthesis involves a complex process with high costs and low yield, our study aimed to construct a recombinant bacterial strain that secretes HD-5 by splicing its gene via overlapping extensions into the nisin-controlled gene expression (NICE) system of *Lactococcus lactis* [19].

## Methods

### Strains and vector

*Lactococcus lactis NZ9000,* the pMD19-T simple plasmid and the pNZ8148-sp vector were obtained by Professor Wei Chen (Jiangnan University, China).

### Construction of the recombinant *L. lactis* strain NZ9000SHD-5

**Fig. 1:**
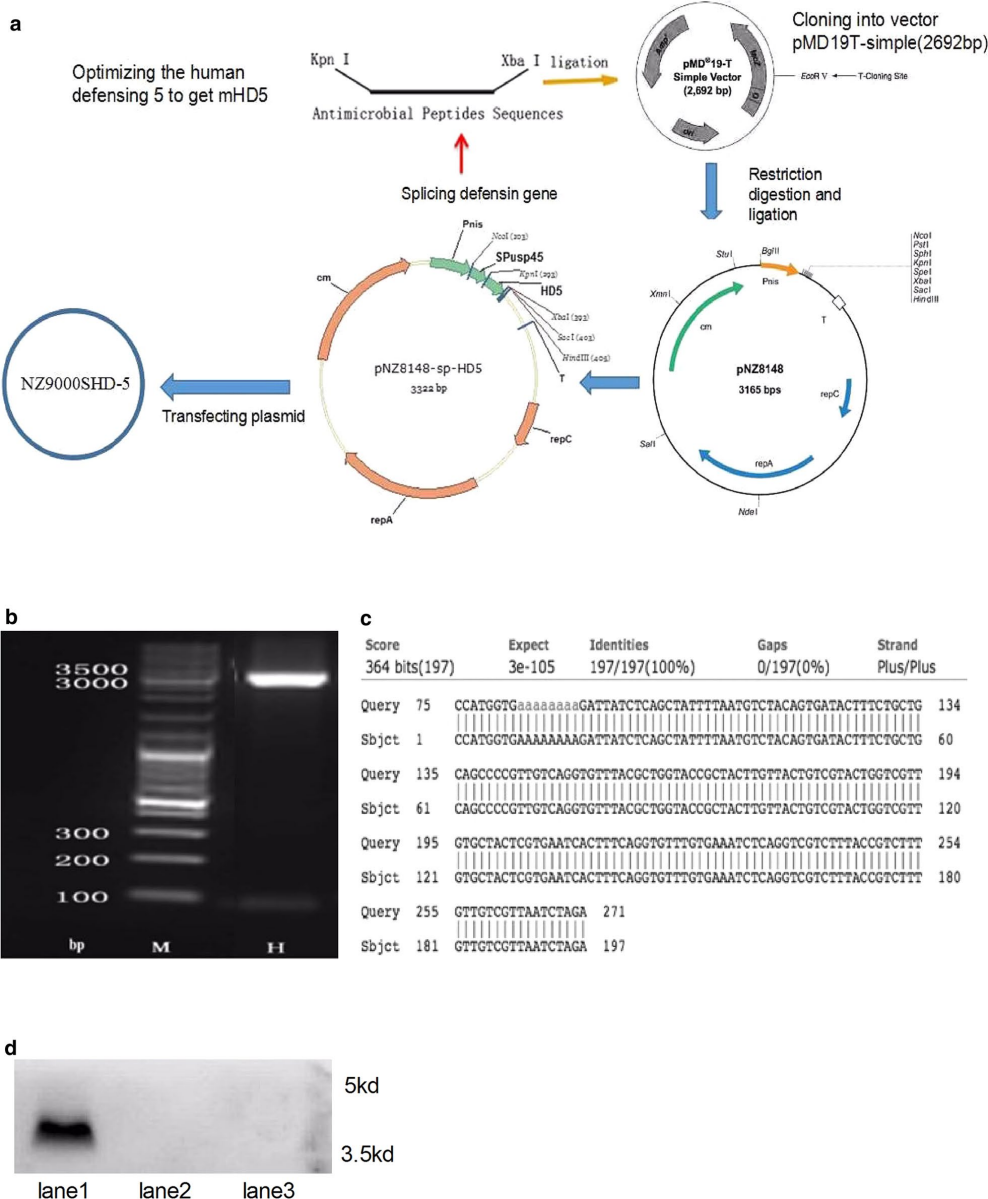
Construction of recombinant *Lactococcus lactis*. **a** The constructing procedure of NZ9000SHD-5. **b** The defensin gene (115 bp) was confirmed to be successfully ligated to the secreting plasmid pNZ8148-sp. **c** Gene sequencing of recombinant NZ9000SHD-5 confirmed the successful construction of the recombinant *Lactococcus lactis*. **d** The expression of defensin in the supernatant was measured by Western Blotting with specific antibodies. (Bar 1: recombinant NZ9000SHD-5 with nisin; Bar 2: recombinant NZ9000SHD-5 without nisin; Bar 3: recombinant NZ9000 with nisin)

We optimized a defensin mHD-5 clone primarily consisting of the mature HD-5 peptide according to the preference codon of *L. lactis*, in order to enhance expression in this microorganism. We obtained the defensin sequences by overlapping extension PCR, and then inserting the restriction enzyme sites KpnI and XbaI into the gene sequences. Finally, we ligated the HD-5 genes to the pMD19-T simple plasmid to construct a recombinant plasmid, which was transfected into *Escherichia coli* Top 10 by chemical conversion. After obtaining positive clones, successful construction of the plasmids was confirmed through enzyme digestion and gene sequencing (Fig. 1a–c). The sequences extracted were ligated to the secretory expression vector pNZ8148-sp, and then transfected into *L. lactis* NZ9000, thus building recombinant *L. lactis.* Next, we extracted the protein from the supernatant of NZ9000SHD-5 and verified the expression of HD-5 by Western blot (Fig. 1d). In this article, NZ9000 designates the recombinant *L. lactis* strain harboring the same vector lacking the defensin gene (pNZ8148-sp).

### Microorganisms and media

The recombinant NZ9000SHD-5 and NZ9000 strains were grown in M17 agar (Hope biotechnology, Qingdao, China) on a plate with 1% d-glucose and 20 μg/ml chloramphenicol for culture at 30 °C and in M17 broth (Hope biotechnology, Qingdao, China) with 1% d-glucose and 20 μg/ml chloramphenicol for enrichment culture at 30 °C. Nisin was added into the bacteria culture tube when the OD_600_ reached 0.5, which implicated *L. lactis* was at its exponential growth phase. The supernatant after 2 h.

### Animal model

Male C57/BL6 mice (6 to 8 weeks old) were obtained from the Laboratory Animal Center of Southern Medical University (Guangzhou, China) and maintained in plastic cages under standard conditions. A diet of standard pellets was provided ad libitum. Acute colitis was induced by oral intake of 3.5% dextran sodium sulfate (DSS) (w/v, molecular mass of 36,000–50,000 Da; MP Biomedicals, Solon, OH) in fresh water ad libitum for 7 days (n = 7/group). No major differences in water consumption were detected among the groups.

The purpose of this study was to investigate the protective effect of the NZ9000SHD-5 strain against inflammation and mucosal lesions in DSS-induced colitis. Mice were divided into four groups: The control group was administered PBS once daily for 7 consecutive days, whereas the three remaining groups were administered 3.5% DSS for 7 days. These DSS-treated groups were also administered the NZ9000 strain, the NZ9000SHD-5 strain or PBS throughout the DSS treatment period. The mice were euthanized on day 8 by cervical dislocation, and blood and tissue samples were collected. Colons were separated from the proximal rectum near their passage under the pelvisternum. The colon length between the ileocecal junction and the proximal rectum, an indicator of disease, was measured and weighed. Some colonic tissue was excised and homogenized in RIPA lysis buffer. Equal amounts of protein (40 µg/lane) were subjected to Western blotting and ELISA. Other colonic tissue samples were subjected to H&E staining as previously described. Remaining tissues were stored at − 80 °C until further analysis.

### Assessment of disease activity

The disease activity index (DAI) was assessed according to a standard scoring system by an investigator who was blinded to treatment protocol. The BW, stool consistency, and OB levels in stool were recorded for 7 days. The assessment of DAI followed the protocol of previous studies [20].

### Histological staining with H&E

Colon tissue was collected for histological analysis. Samples were fixed in 4% paraformaldehyde for 48 h and then dehydrated with a graded alcohol series. Afterwards, the tissues were embedded in paraffin and sliced into 5 μm-thick sections. For each sample, the sections were stained with H&E and mounted with Permount (Thermo Fisher Scientific, Philadelphia, PA). Mucus-containing cells were stained a purple-red color.

### Measurement of cytokine levels

The concentrations of IL-6, TNF-α, and IL-1β in the colon and supernatants were quantified using ELISA kits following the manufacturer’s protocols (MultiSciences Biotech, China).

### mRNA analysis using real-time PCR

Total RNA was isolated from either intestinal issues or cells using TRIzol (TaKaRa, Japan) and mRNA expression was measured using PrimeScript™ First-strand cDNA Synthesis Kits (Takara, Japan). The PCR primer sequences were designed using Primer Premier 5.0 and are listed in Table 1. The 2^−ΔΔCt^ method was used to measure the expression levels of target genes with GAPDH as an internal control. We calculated the ΔCt and ΔΔCt values as follows: ΔCt = Ct (target gene) − Ct (housekeeping gene); ΔΔCt = ΔCt (treatment) − ΔCt (control). The 2^−ΔΔCt^ variations served as a surrogate measure of gene expression changes.

**Table 1:** Primers used for RT-PCR

Gene	Primer sequences	Product size (bp)	GenBank accession no.	Species
IL-6	F:GAGTCACAGAAGGAGTGGCTAAGGA	106	NM_031168.1	Mouse
	R:CGCACTAGGTTTGCCGAGTAGATCT			
TNF-α	F:GCATGGTGGTGGTTGTTTCTGACGAT	99	NM_010851.2	Mouse
	R:GCTTCTGTTGGACACCTGGAGACA			
IL-1β	F:CTCGTGCTGTCGGACCCAT	343	NM_008361.4	Mouse
	R:CAGGCTTGTGCTCTGCTTGTGA			
Zo-1	F:TCATCCCAAATAAGAACAGAGC	198	XM_006540786.1	Mouse
	R:GAAGAACAACCCTTTCATAAGC			
Occludin	F:AAGCAAGTGAAGGGATCTGC	204	NM_001205255.1	Mouse
	R:GGGGTTATGGTCCAAAGTCA			
GAPDH	F:CAACGGCACAGTCAAGGCTGAGA	112	NM_017008.3	Mouse
	R:CTCAGCACCAGCATCACCCCAT			
Zo-1	F:AGAGGAAGCTGTGGGTAACG	320	NM_001301025.1	Human
	R:TCCTTCAGCTGGTCCTCCTT			
Occludin	F:CTCCCTGGCACCGTTGG	548	NM_001205254.1	Human
	R:GGCCAACATGAAGCCCTTTG			
β-Actin	F:CTCGCCTTTGCCGATCC	258	NM_001101.3	Human
	R:GGGGTACTTCAGGGTGAGGA			

### Enzyme activity measurements

MPO activity was assessed using ELISA kits following the manufacturer’s instructions. Results were expressed in arbitrary units per 100 mg tissue.

### Cell culture

RAW264.7 and Caco-2 cells were bought from ATCC. Cultures were performed respectively in RPMI1640 medium or DMEM in a 6 well plate (NEST Biotechnology, Wuxi, China), and both media were supplemented with 10–20% heat-inactivated FBS, 50 U/ml penicillin and 50 U/ml streptomycin (Invitrogen, USA) in a 37 °C incubator containing 5% CO_2_. RAW264.7 cells were trypsinized and resuspended at 1 × 10^6^ cells/ml for the subsequent determination of cytokine gene expression. For the lipopolysaccharide (LPS) experiments, cells were treated with 1.0 µg/ml LPS (Sigma, USA) from *Escherichia coli* 0:55:B55 diluted in ddH_2_O for 12 h. In the NZ9000 and NZ9000SHD-5 groups, RAW264.7 cells were treated with LPS and supernatants from NZ9000 or NZ9000SHD-5 cells for 12 h. Caco-2 cells were plated at a density of 1 × 10^6^ cells/ml on collagen-coated permeable polycarbonate membrane Transwell supports with 0.3 µm pores (Corning, USA) and were grown as monolayers for subsequent measurement of TJ gene and protein expression.

### Measurement of transepithelial electrical resistance

Caco-2 cells were cultured at a density of 5 × 10^4^/ml in Transwell chambers for 2 weeks to mimic the monolayer of the epithelial barrier. The NZ9000SHD5 and NZ9000 groups were pre-incubated with 200 µl of the corresponding supernatants for 4 h, and each group was then administered 3.5% DSS and supernatants as appropriate. After establishment of the Caco-2 cell monolayers, transepithelial electrical resistance (TEER), an indicator of TJ permeability to ionic solutes, was measured at 1 h, 2 h, 4 h, 8 h, 12 h, and 24 h using a Millicell-ERS voltohmmeter (Millipore, USA) according to manufacturer instructions.

### Measurement of fluorescein isothiocyanate (FITC) intensity

The intestinal permeability to 4000-Da FITC-dextran (DX-4000-FITC, Sigma, USA) was determined as previously described. Following a 12-h fasting period, the mice were administered FITC-dextran via intragastric infusion (60 mg/100 g BW, 20 mg/ml). After 4 h, blood was collected and centrifuged at 3000*g* and 4 °C for 10 min, and the resulting plasma layer was diluted in an equal volume of PBS. For the in vitro experiments, the Caco-2 cell monolayer was treated with 3.5% DSS prior to FITC-dextran administration (1 mg/ml). The in vivo and in vitro FITC-dextran concentrations in the serum and culture media were analyzed with a fluorescence spectrophotometer (Thermo, USA) at an excitation wavelength of 485 nm and an emission wavelength of 525 nm. Standard curves were obtained by diluting FITC-dextran in non-treated plasma diluted with PBS.

### Western blot analysis

Total protein from cells and colon tissues was extracted using a Whole Protein Extraction kit (KeyGen BioTech, China) according to the manufacturer’s instructions. Equal amounts of protein (40 µg) from each specimen were loaded onto a 10% polyacrylamide gel and subjected to SDS-PAGE, followed by transfer onto a polyvinylidene difluoride (PVDF) membrane. The membrane was blocked with 5% skim milk for 1 h, after which specific monoclonal antibodies targeting p65, p-p65, IκBα, p-IκBα, ZO-1, occludin or GAPDH were applied. The target proteins were detected with HRP-conjugated secondary antibodies for 1 h, and after three washes, the specific bands were visualized using an enhanced chemiluminescence (ECL) detection kit (Fudebio, Hangzhou, China).

### Immunofluorescence staining of cells

After the cells were grown in chamber slides, they were washed 3 times with PBS and fixed with 4% paraformaldehyde for 20 min at room temperature. The cells were blocked with 1% BSA at room temperature for 1 h and washed again with PBS. Next, the cells were incubated overnight at 4 °C with antibodies targeting occludins and ZO-1 (1:50 dilution, Abcam, Cambridge, UK). After several additional washes, the slides were incubated again for 30 min with either FITC-conjugated or Cy3-conjugated secondary antibodies. Nuclei were counter-stained with diamidino-2-phenylindole (DAPI) (Sigma, USA), and the slides were observed using a fluorescence microscope (Olympus, Japan).

### Statistical analysis

All statistical analyses were performed using SPSS software version 19.0. Data are presented as mean ± standard deviation. A homogeneity test for variance was performed first; if the data followed a normal distribution and showed homogeneity of variance, ANOVA with the post hoc LSD test was performed. *P *< 0.05 was considered statistically significant.

## Results

### NZ9000SHD-5 ameliorated colon injury and inflammatory symptoms in DSS-induced UC model mice

**Fig. 2:**
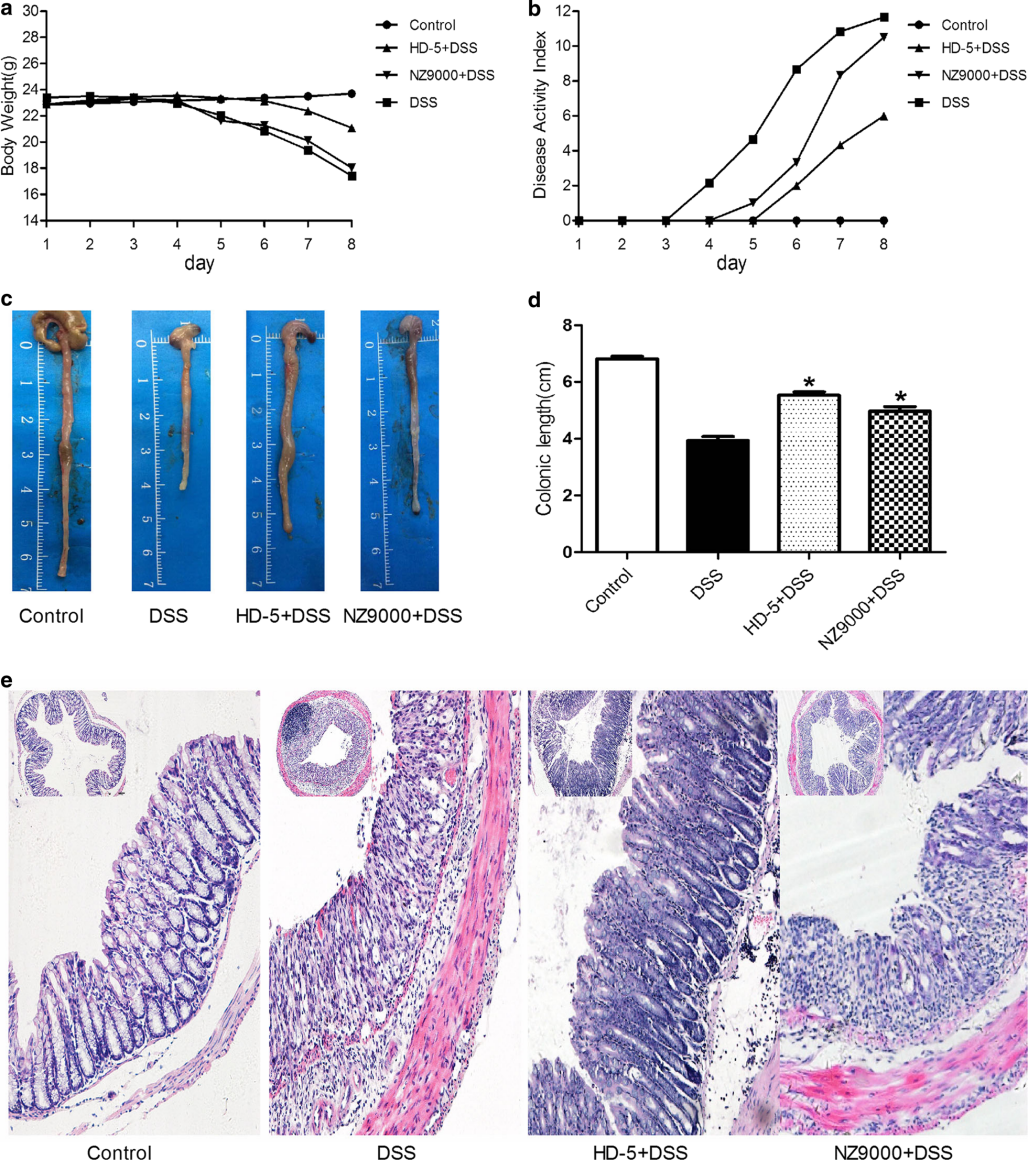
NZ9000SHD-5 reduced the susceptibility of mice to DSS-induced colitis. **a** Body weight measures, day 0 to day 7. **b** The disease activity index included body weight loss, stool consistency and the occult blood test. **c**, **d** Differences in colon length between groups and strip statistical analysis diagram. **e** HE staining sections. Differences between groups were determined by ANOVA followed by the post hoc LSD test (n = 6). *p < 0.05 as compared to the DSS group. ^*#*^*p *< 0.05 as compared to the NZ9000 group

To characterize the effect of NZ9000SHD-5 on inflammation, mice from each treatment group were provided drinking water supplemented with 3.5% DSS for 7 days. The animal model showed successful DSS-induced damage to the colonic mucosal barrier, leading to significant inflammation and loss of body weight (BW). BW loss, fecal consistency and occult blood (OB) scores constitute the Disease Activity Index (DAI), an important metric for the assessment of colitis severity. BW loss was recorded daily; from days 5 to 8, the DSS group showed a greater BW reduction from baseline than the control group, whereas NZ9000SHD-5 group showed a smaller decrease in BW over the same period (Fig. 2a). Meanwhile, disease progression and clinical scores were dramatically different in the NZ9000SHD-5 and DSS groups. The control group consistently showed a DAI score of 0, as these mice did not exhibit any inflammatory symptoms. Both the NZ9000 and DSS groups had peak DAI scores on day 8, yet NZ9000SHD-5 group had lower scores as these mice had firmer stools and less OB. Thus, we surmised the recombinant *L. lactis* strain expressing HD-5 induced a significant reduction in the DAI scores of DSS-treated mice (Fig. 2b).

Colon length is another accepted index of colonic inflammation severity. Consistent with the DAI results, the DSS group showed shorter lengths than the control and NZ9000SHD-5 groups (Fig. 2c, d). On the other hand, histological analysis is the most direct method for evaluating colon tissue damage and treatment efficacy. We observed more severe pathological damage as well as much greater neutrophil and macrophage infiltration in the DSS group than the NZ9000SHD-5 group. Compared to controls, the DSS group showed extensive colonic ulcerations and intestinal crypt damage, along with increased mucosal infiltration of granulocytes and mononuclear cells. Compared to the DSS group, mice treated with the NZ9000SHD-5 strain presented reduced histological evidence of DSS-induced colitis (Fig. 2e). Therefore, we hypothesize NZ9000SHD-5 may attenuate the clinical symptoms of colitis.

### NZ9000SHD-5 inhibited the infiltration of pro-inflammatory cytokines in DSS-induced colitis mice

**Fig. 3:**
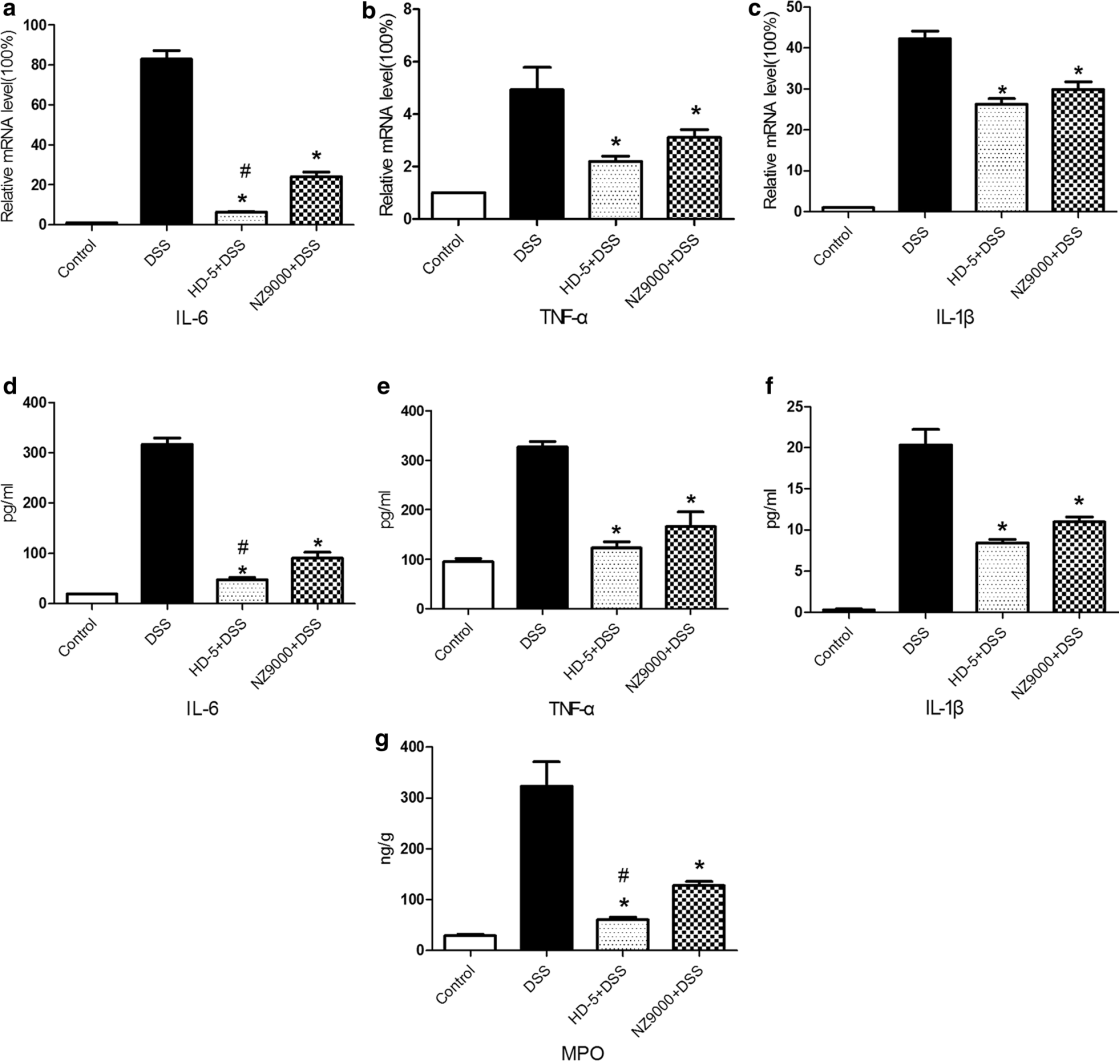
Protective effect of NZ9000SHD-5 on intestinal inflammation in DSS-induced colitis mice. **a**–**c** Quantitative RT-PCR and **d**–**f** ELISA for IL-6, TNF-α and IL-1β in colon tissues. **g** The MPO, as an indicator of inflammatory infiltration, was shown in graft. Differences between groups were determined by ANOVA followed by the post hoc LSD test (n = 6). *p < 0.05 as compared to the DSS group. ^*#*^*p *< 0.05 as compared to the NZ9000 group

To assess the protective effect of NZ9000SHD-5 on DSS-induced colitis, we evaluated the protein levels and gene expression of common pro-inflammatory cytokines TNF-α, IL-6, and IL-1β in colon tissue. Compared to controls, the DSS group showed higher mRNA expression of TNF-α, IL-6 and IL-1β. In contrast, mRNA levels of TNF-α, IL-6 and IL-1β were significantly decreased in the NZ9000 and NZ9000SHD-5 groups. Furthermore, there was a significant difference in IL-6 mRNA expression between the NZ9000 and NZ9000SHD-5 groups (*p *< 0.05) (Fig. 3a–c).

The DSS group showed significantly higher levels of TNF-α, IL-6 and IL-1β in the colon in comparison to controls. Moreover, the NZ9000 and NZ9000SHD-5 groups presented remarkably reduced DSS-induced production of TNF-α, IL-6 and IL-1β in comparison to the DSS group. Consistent with the significant effects of NZ9000SHD-5 on the expression of mRNA corresponding to pro-inflammatory cytokines, there was also a significant difference in IL-6 protein levels between the NZ9000 and NZ9000SHD-5 groups (*p *< 0.05) (Fig. 3d–f).

The infiltration of inflammatory cells, especially neutrophils and macrophages, in colonic tissue are correlated with the severity of inflammation. Myeloperoxidase (MPO) can be utilized as an indicator of neutrophil infiltration. In this study, we ascertained a significantly higher MPO levels in tissue samples from the DSS group than in the control group. Administration of either the NZ9000 or NZ9000SHD-5 strains decreased MPO activity, with the NZ9000SHD-5 strain showing a greater effect (*p *< 0.05) (Fig. 3g). Together, these results indicate a protective effect for the NZ9000SDH-5 strain in attenuating colonic inflammation associated with DSS-induced colitis.

### NZ9000SHD-5 improved the intestinal mucosal barrier function in vivo

**Fig. 4:**
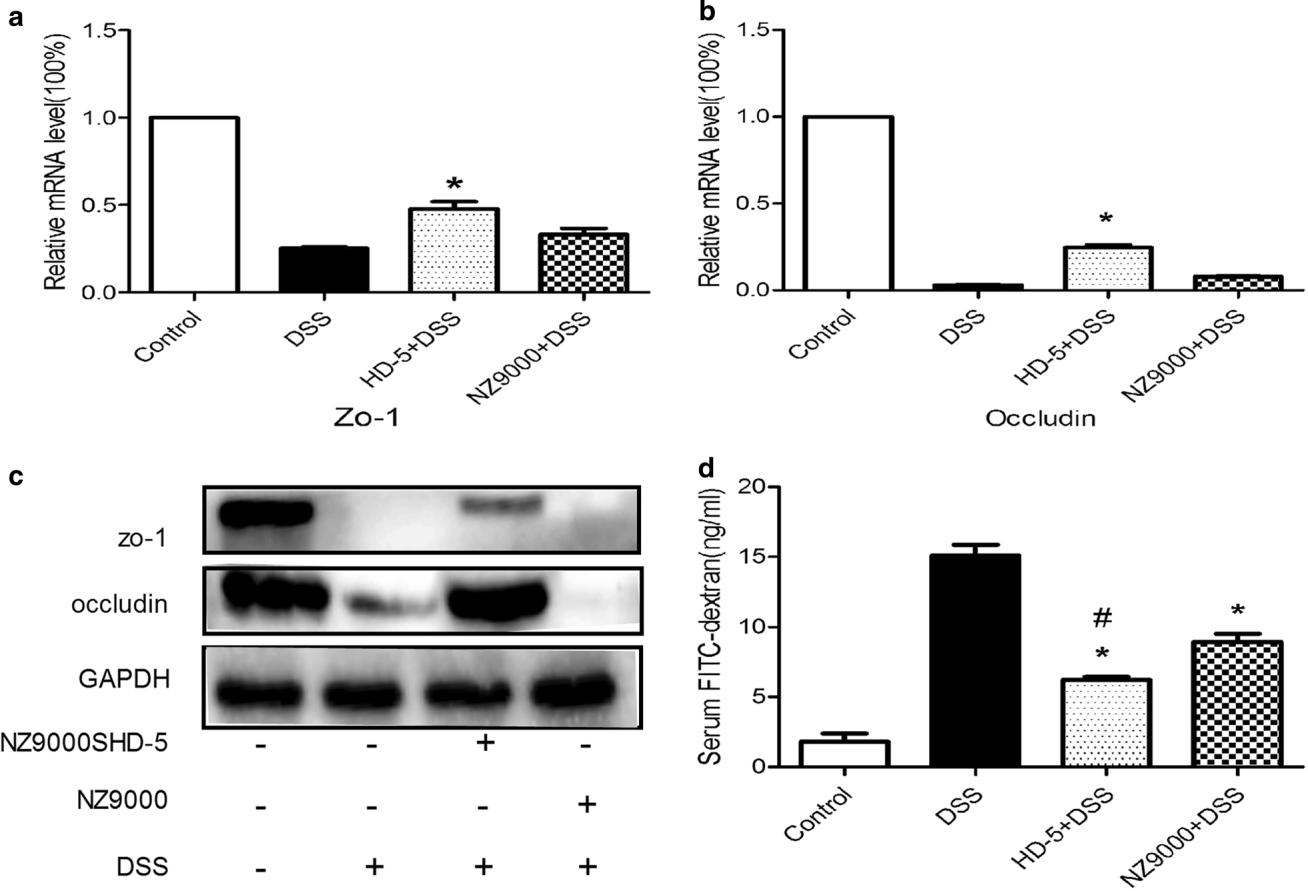
Protective effect of NZ9000SHD-5 on tight junction structure and function in vivo. Relative mRNA expression of **a** ZO-1 and **b** Occludin. **c** Protein expression levels of ZO-1 and Occludin in colon tissues. **d** Quantification of serum FITC-dextran. Differences between groups were determined by ANOVA followed by the post hoc LSD test (n = 6). *p < 0.05 as compared to the DSS group. ^*#*^*p *< 0.05 as compared to the NZ9000 group

Dysfunction of the gut barrier contributes to the pathogenesis of intestinal disease. To assess the effect of NZ9000SHD-5 in this aspect, we focused on the structure of TJ. The expression levels of ZO-1 and occludins were compared in vitro and in vivo using RT-PCR and Western blotting. And we found that mRNA expression levels of ZO-1 and occludins were lower in the DSS group than in the control group, and administration of NZ9000SHD-5 increased the expression of all examined genes (Fig. 4a, b). Furthermore, expression of TJ proteins was increased in the NZ9000SHD-5 group, whereas the mice treated with DSS alone showed a significant decrease in TJ protein expression (Fig. 4c).

To further assess the protective effect of NZ9000SHD-5 on gut barrier function, intestinal permeability was assessed using FITC-dextran. The levels of FITC-dextran in serum were significantly increased in DSS-treated mice, suggesting deterioration of the intestinal barrier and increased paracellular permeability. Administration of NZ9000SHD-5 or NZ9000 to DSS-treated mice resulted in decreased permeability to FITC-dextran compared with DSS treatment alone, and the effect of NZ9000SHD-5 was more obvious than that of NZ9000 (Fig. 4d). Therefore, we concluded NZ9000SHD-5 may have a powerful role in preserving intestinal barrier structure and function.

### NZ9000SHD-5 inhibited pro-inflammatory cytokines production and protects intestinal barrier integrity in vitro

**Fig. 5:**
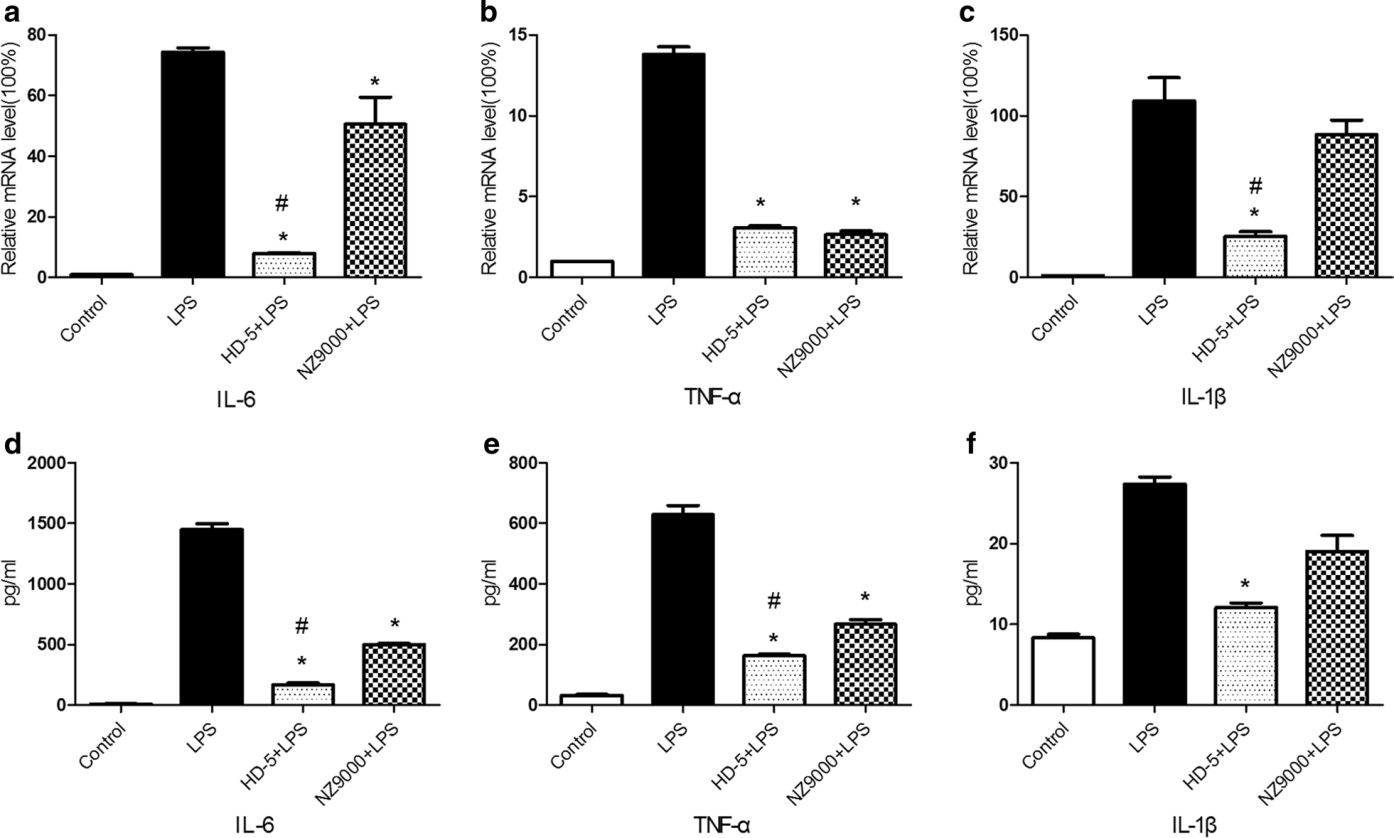
NZ9000SHD-5 inhibited the secretion of pro-inflammatory cytokines in LPS-induced RAW.264.7 cells. RAW264.7 cells were incubated with LPS (1 ug/ml) for 12 h. Expression of **a** IL-6, **b** TNF-α, **c** IL-1βmRNA was assessed with real-time PCR. **d**–**f** The levels of inflammatory cytokines in the supernatant were assessed by ELISA. Differences between groups were determined by ANOVA followed by the post hoc LSD test (n = 4). *p < 0.05 as compared to the DSS group. ^*#*^*p *< 0.05 as compared to the NZ9000 group

To confirm the inhibitory effect of NZ9000SHD-5 on the expression of inflammatory molecules, as well as its protective effect on gut barrier integrity, we performed an in vitro experiment using RAW264.7 and Caco-2 cells. As shown in Fig. 5, we measured the expression of IL-6, TNF-α and IL-1β mRNA in cells through RT-PCR (Fig. 5a–c), and their corresponding protein levels in cell supernatants (Fig. 5d–f) through ELISA. Both types of indicators were higher in cells stimulated with LPS (1 μg/ml) than in untreated cells, but these changes were inhibited by pretreatment with supernatants from NZ9000SHD-5 cultures (*p *< 0.05).

**Fig. 6:**
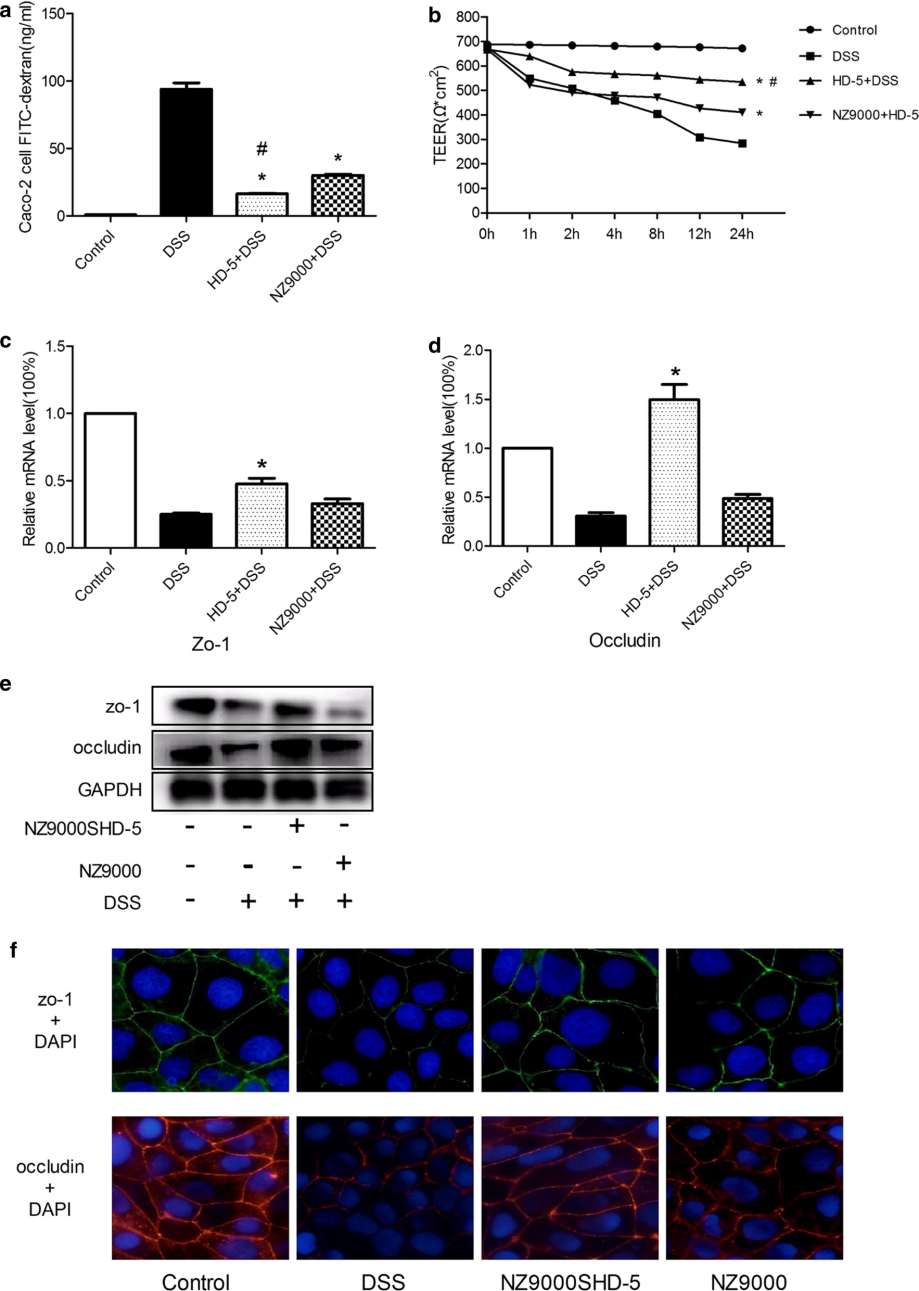
Protective effect of NZ9000SHD-5 on intestinal tight junctions in vitro. **a** Changes in TEER in Caco-2 cell monolayers exposed to different treatment was determined at 1, 2, 4, 8, 12 and 24 h. **b** FITC-dextrum quantification was compared between groups. Relative changes of gene expression of **c** zo-1 and **d** occludin were measured by real-time PCR. **e** Expression of TJ-related proteins in Caco-2 cells. **f** Cytoskeleton integrity, with zo-1 shown in green and occludin in red. Differences between groups were determined by ANOVA followed by the post hoc LSD test (n = 6). *p < 0.05 as compared to the DSS group. ^*#*^*p *< 0.05 as compared to the NZ9000 group

Meanwhile, to elucidate the molecular mechanisms underlying the beneficial effects of NZ9000SHD-5 in intestinal barrier function, we incubated a Caco-2 cell monolayer with 3.5% DSS either alone or together with NZ9000SHD-5 or NZ9000 culture supernatant. The FITC-dextran experiment was performed on these Caco-2 monolayers. The results showed that administration of NZ9000SHD-5 culture supernatant resulted in lower permeability to FITC-dextran than DSS treatment alone (Fig. 6a). We also measured trans-epithelial electrical resistance (TEER) on the monolayers. TEER was decreased at 2 h and reached its nadir at 4 h. Cells treated with DSS and NZ9000SHD-5 culture supernatant showed slightly reduced TEER at 2 h and presented relatively stable values at 8 h (Fig. 6b). By the end of the study, the DSS-treated Caco-2 monolayer showed a significantly lower average TEER than the NZ9000SHD-5 culture supernatant-treated cells (*p *< 0.05).

We measured mRNA and protein expression levels of ZO-1 and occludin in Caco-2 cells in the control group, DSS group, DSS & NZ9000SHD-5 group, and DSS & NZ9000 group. These indicators were significantly higher in the DSS & NZ9000SHD-5 group than in the DSS group, and there was only a few nonspecific effects in the DSS & NZ9000 group (Fig. 6c–e). To more clearly visualize these changes, we performed immunofluorescence for ZO-1 and occludin in Caco-2 cells. The results showed that ZO-1 (green) and occludin (red) were mostly observed on the cell membrane, constructing a clear and intact cytoskeletal network in the control group. In the DSS group, the expression of these TJ proteins was reduced, and the cytoskeletal network was destroyed. In contrast, administration of NZ9000SHD-5 culture supernatant attenuated this disruption more obviously when compared with NZ9000 group (Fig. 6f).

### NZ9000SHD-5 culture supernatant suppressed the NF-kB signaling in vitro

**Fig. 7:**
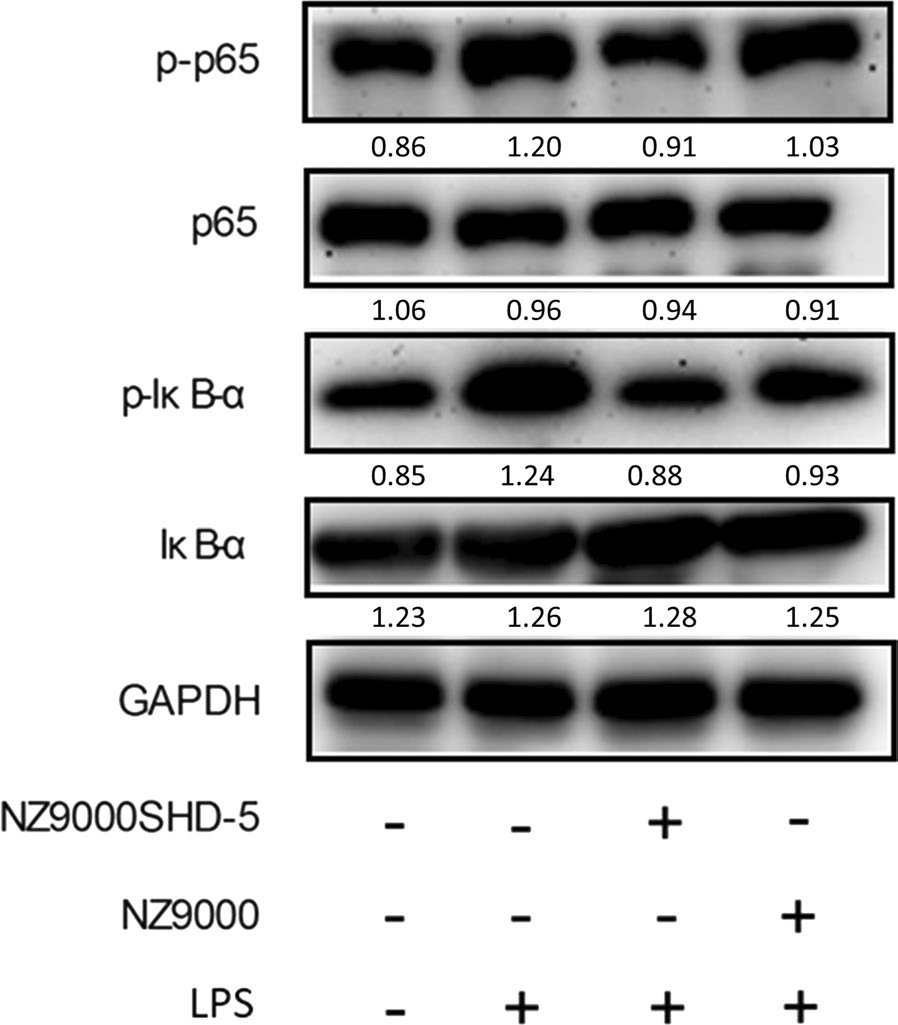
NZ9000SHD-5 inhibited NF-κB translocation in LPS-induced RAW264.7 cells. Phosphorylated and total protein levels of p65, IκB, and GAPDH were determined by specific phosphorylated and total protein antibodies. LPS (1 μg/ml) increased the phosphorylation of p65 and IκB-α, while treatment with NZ9000SHD-5 inhibited this effect

Western blotting showed that, in comparison to non-treatment, exposure to LPS in RAW264.7 cells elevated the levels of phosphorylated p65 NF-κB, a key factor in the NF-κB signaling pathway; as well as IκBα, the signaling molecule upstream of NF-κB translocation (Fig. 7). Indeed, treatment of these cells with supernatants from NZ9000SHD-5 cultures appeared to suppress the expression of pro-inflammatory cytokines, and this effect appeared to be associated with NF-κB translocation.

## Discussion

Because the intestine is constantly exposed to a large variety of microorganisms, preservation of the integrity of the epithelial layer is essential to keep pathogens in the lumen and maintain the balance of nutrient exchange [21]. Compromise of this epithelium facilitates the development of diseases such as IBD [22, 23]. Although the pathogenesis of IBD has not been fully elucidated, colonic epithelial damage and mucosal barrier dysfunction are critical targets to be addressed in order to ameliorate the symptoms of IBD and maintain disease remission [24]. Research suggests these alterations favor extraintestinal translocation of commensal bacteria, promoting immune activation and intestinal inflammation in patients with IBD [25, 26]. Human α-defensins participate in the preservation of gut barrier function and immunomodulation in this context. Attenuated expression of defensins undermines host immunity and may promote inflammation [27, 28]. A previous study demonstrated a deficiency of human α-defensin expression in subjects with IBD [29]; and defensins released by Paneth cells appear to be critical in the pathogenesis and progression of IBD [30, 31]. Besides, human α-Defensin-5 could be used as a potential candidate biomarker to molecularly differentiate Crohn’s colitis (high levels of Human α-Defensin-5) from UC (low levels of Human α-Defensin-5) [32]. In this study, we used the typical DSS mouse model of colitis to test the effects of α-defensin administration in the pathophysiology of colitis. Results revealed that NZ9000SHD-5 alleviated colonic damage and clinical symptoms, and reduced the levels of inflammatory cytokines and neutrophil infiltration. Recombinant defensin also protected gut barrier function, as this treatment alleviated the DSS-induced reduction in TJ protein expression, and preserved colonic paracellular permeability. These results were consistent with previous research which proved that HD-5 feeding attenuated ethanol and colitis-induced dysbiosis, mucosal inflammation, damage of epithelial integrity and endotoxin translocation in the small intestine and colon [31].

The DSS-induced mouse model was used to simulate the acute colitis experienced by patients with ulcerative colitis [33, 34]. This model presents clinical symptoms such as diarrhea, hematochezia, BW loss and shortening of the colon, along with histological changes including ulcers, inflammatory mucosal lesions and infiltration of inflammatory cells [35, 36]. Exposure to the NZ9000SHD-5 strain effectively alleviated the symptoms of DSS-induced inflammation, such as BW loss, colon shortening, resulting in lower DAI scores.

Infiltration of inflammatory cells is a common histological feature in IBD which has been associated with the production of pro-inflammatory cytokines and chemokines, disruption of intestinal epithelial TJs, gut dysbiosis and defection in anti-microbial peptide secretion by Paneth cells [37, 38]. Levels of MPO, a peroxidase enzyme present in the azurophilic granules of neutrophils and monocytes, are proportional to the density of neutrophils in lesions; thus, this parameter has been used as an indicator of neutrophil infiltration [39]. Administration of the recombinant NZ9000SHD-5 strain significantly reduced MPO activity, with hematoxylin and eosin (H&E) staining showing reduced infiltration and milder mucosal and overall damage in colonic tissue.

An imbalance between pro-inflammatory and anti-inflammatory cytokines has been implicated in the development of inflammation in patients with IBD, with upregulation of IL-6, TNF-α and IL-1β playing a central role in this context. Indeed, increased levels of serum IL-6 and TNF-α are typically detected in IBD patients; and anti-IL-6R antibodies and TNF-α-blocking agents could be powerful therapeutic alternatives for achieving remission in IBD [40, 41]. In our study, levels of these pro-inflammatory cytokines were increased in the DSS-treated group, whereas NZ9000SHD-5 administration exerted an distinct anti-inflammatory effect by suppressing pro-inflammatory cytokine expression compared with DSS-treated group, it is worth noting that NZ9000SHD-5 treatment could decreased IL-6 and MPO expression significantly compared with NZ9000 group, which confirmed that HD-5 had the effect of inhibiting intestinal inflammation.

NF-κB is a transcription factor that controls the expression of genes involved in the inflammatory response. Pro-inflammatory cytokines such as IL-6, TNF-α and IL-1β are transcriptionally regulated by NF-κB, and increased expression of this factor has been implicated in the pathogenesis of IBD [42]. The NF-κB/Rel complex is mainly composed of RelA (p65) and NF-κB1 (p50) subunits. These dimers are sequestered in inactive cytoplasmic complexes by the inhibitory IκB proteins. The canonical IκBs include IκBα, IκBβ and IκBε, with IκBα as the primary and most important regulator of NF-κB in stimulating the immune response. When responding to extracellular stimuli such as pro-inflammatory cytokines or LPS, the IκB proteins (IκBα and IκBβ) are phosphorylated by the IκB kinase complex (IKK) at conserved N-terminal residues. Phosphorylated IKBα is rapidly polyubiquitinated, exposing the nuclear localization sequence of NF-κB and allowing subsequent nuclear translocation, where it then promotes the expression of pro-inflammatory genes. Suppressing this signaling pathway may be an effective method in the treatment of colitis. In our study, Western blotting revealed the expression of phosphorylated p65 in response to LPS was attenuated by pretreatment with NZ9000SHD-5 culture supernatant. Similarly, phosphorylation of IKBα was suppressed by NZ9000SHD-5 culture supernatant. This suggests the NZ9000SHD-5 strain downregulates the expression of pro-inflammatory cytokines by suppressing the NF-κB signaling pathway.

Epithelial TJ integrity is vitally important to defend a “leaky gut,” which would allow harmful molecules such as LPS to permeate the intestinal epithelium and enter the peripheral blood. Alterations occurring to the barrier function are known to contribute to IBD. TJ molecules are crucial for maintaining the integrity of the intestinal epithelial barrier and permeability [6]. Our data showed the protective effect of the NZ9000SHD-5 strain on intestinal barrier function occurs through inhibiting the DSS-induced reduction in TJ protein expression and its subsequent increase in intestinal permeability.

There are several limitations of our study. We compared the differences among the treatment groups and demonstrated the defensin secreted from the recombinant bacterial strain had a notable effect. However, the specific characteristics of the colonization achieved by this recombinant *L. lactis* strain should be further detailed. In addition, although we ascertained the immunomodulatory effect of NZ9000SHD-5 to be related to the NF-κB signaling pathway, the exact mechanisms that leaded to the restoration of disrupted epithelial barrier function remained a key subject for investigation in further research. And nevertheless, other factors except for defensins released by NZ9000SHD-5 strain could also play a beneficial role against IBD, we need to further purify the proteins produced by it in subsequent experiments.

## Conclusions

Our study demonstrates the recombinant NZ9000SHD-5 strain protects intestinal mucosal barrier function by maintaining the integrity of the TJ network and regulating the immunological response via suppression of NF-κB signaling pathway, which results in decreased expression of pro-inflammatory cytokines. Therefore, exogenous administration of defensins may be a novel and potentially effective treatment for patients with IBD.

## Data Availability

The datasets used and analyzed in this study will be made available by the authors under reasonable circumstances.

## Abbreviations

*L. lactis*: *Lactococcus lactis*
DSS: Dextran sodium sulfate
HD-5: Defensin-5
TNF-α: Tumor necrosis factor-α
IL-6: Interleukin-6
IBD: Inflammatory bowel disease
UC: Ulcerative colitis
CD: Crohn’s disease
TJ: Tight junction
NICE: Nisin-controlled gene expression
DAI: Disease Activity Index
BW: Body weight
OB: Occult blood
MPO: Myeloperoxidase
TEER: Trans-epithelial electrical resistance
H&E: Hematoxylin and eosin
NF-κB: Transcription factor-kappa B
IKK: IκB kinase complex
LPS: Lipopolysaccharide
FITC: Fluorescein isothiocyanate
PVDF: Polyvinylidene difluoride
ELISA: Enzyme-linked immunosorbent assay
ECL: Enhanced chemiluminescence
DAPI: Diamidino-2-phenylindole
